# Estimates for the Prebiotic Reaction Volume Necessary for the Origin of Life

**DOI:** 10.3390/life16101708

**Published:** 2026-10-12

**Authors:** David A. Juckett

**Affiliations:** Department of Pharmacology & Toxicology, Michigan State University, East Lansing, MI 48842, USA; juckett@msu.edu

**Keywords:** origin of life, panspermia, complexity, CHNOPS, prebiotic reaction volume, time, rate of evolution

## Abstract

The prebiotic chemistry necessary for the development of life requires a low-probability combination of steps to reach the complexity of a living cell. An unanswered question is how big the reaction space must be to favor this process, if it is driven by stochastic forces. The space must be large enough and diverse enough to provide reaction centers as well as storage reservoirs to hold chemical intermediates. Using the carbon footprint of various lifeforms on Earth and their average cell masses, the number of cells alive today in various taxa can be calculated. It is shown that as cells increase in mass and complexity, they occupy progressively smaller shares of the biosphere and exist in smaller numbers. Conversely, the basic units of life increase in number as complexity decreases. Following this trajectory to the starting atomic elements of life yields a count of the CHNOPS needed to enable prebiotic chemistry to begin. Estimates of the starting volume of water necessary to contain this number of elements approaches the volume of Earth’s oceans. Furthermore, assigning the cell counts of various taxa to their times of first appearance on Earth yields a linear regression that can be extrapolated back in time to a point when only CHNOPS existed. This occurs at ~9.0 Gyr before the present. The implications of these findings are discussed, with a particular focus on a world existing 9 Gyr ago, with characteristics similar to Europa, with a large, protected water environment that can store chemical intermediates over a long time and thereby support combinatoric prebiotic chemistry.

## 1. Introduction

Prebiotic chemistry is the study of the chemical reactions that create structures and pathways leading ultimately to living cells. To undertake such studies, the many unknowns of deep time must be estimated and important limits for environmental constraints need to be defined using the geophysics and geochemistry of the early universe and solar system [1]. As more ways of probing these limits are developed, the constraints can be narrowed. Nevertheless, at the very minimum, prebiotic chemistry requires atomic starting materials, suitable energy fluxes, and favorable conditions for temperature, liquid water, pH, catalysts for reactions, and time. Furthermore, to transform an ever-growing constellation of products into ever-growing and evolving biochemical constructs, there must also be memory—that is, a way to store these growing products and constructs without degradation. If the process is stochastic in nature, the memory storage must be vast. Therefore, the combined reaction and memory volume is an environmental constraint that must be addressed and is the topic of this communication.

In a previous paper [2], various components associated with the complexity of life were identified for taxa spanning ~3.5 Gyr of life on Earth. Each of these components exhibited linearly increasing complexity as time progressed from early Earth until the present day. Extrapolating the linear regressions backward in time, until simpler complexity versions of each component were reached, yielded evidence of an aggregate minimal complexity occurring around 8.5 Gyr ago, a conclusion also reached by Sharov and Gordon [3]. Each of the complexity components examined were intrinsic to life and, by inference, prebiotic chemistry. None of the components, however, addressed the environmental constraint of reaction and memory chamber size necessary for successful prebiotic chemistry.

Reaction and memory volumes have received little attention in the origin-of-life field. This may be due to an implicit bias derived from the assumption that life must have started on Earth, and, thus, it likely would have occurred in the available enclaves of deep-sea vents, shallow tidal pools, asteroid impact craters, hot springs, clay beds, etc. [4,5,6,7,8,9,10,11,12,13,14,15,16]. Because of their small volumes, each of these environments must embody the implicit assumption that prebiotic chemistry followed a narrow path to functional biochemistry, organization, genetic memory, reproduction, and plasticity without the need for both the massive possibilities of combinatoric chemistry and all the reactions that lead to dead-end constructs. Even combining all these proposed enclaves [17] does not create a volume that addresses the memory and time issues adequately. If one assumes combinatoric chemistry, then a “diffuse root tree of life” [18] is the more likely route to life. Such chemistry may be impossible, within a reasonable time, in the small enclaves available on early Earth. Therefore, an estimate of the reaction space seems appropriate to determine if alternatives to Earth are favored.

One method with which to estimate the reaction and memory volume for successful prebiotic chemistry is to calculate the water volume needed to house a sufficient quantity of the basic elements of life under the assumption that initial chemistry occurred in a stochastic manner. The volume must be large enough to allow for the creation of the many intermediates needed for the further anabolic and catabolic reactions that favored complexity growth. As reaction products and constructs grew in complexity, their absolute numbers would have decreased as elements consolidated into molecules and molecules into polymers. This is a natural consequence of energy, entropy, and mass limits in any self-contained environment. Such reductions would have continued throughout the steps of prebiotic chemistry, cell formation, and evolution. The portion of this process that occurred in living organisms can be examined using current data. It can be shown, using carbon biomasses of various taxa groups [19], that as cells increase in complexity, they occupy progressively smaller shares of the biosphere and exist in smaller numbers. Calculating the number of cells in each of these taxa shares yields a pyramid of life. At the bottom of this pyramid lie the basic elements of life, CHNOPS (Carbon Hydrogen Nitrogen Oxygen Phosphorus Sulfur). Determining the quantity of CHNOPS elements then enables a calculation of the volume that they must occupy under various assumptions. The conclusion is that a volume representing a significant percent of Earth’s total ocean mass is required. This is discussed in the context of the constraints of the time and probable temperature needed for a memory chamber.

The taxa cell counts can also be plotted as [log(cell count)] vs. [time of first appearance]. This reveals a linear regression that can be extrapolated back into the past when prebiotic constructs were likely to have been the most complex units present. The extrapolation intersects the basic CHNOPS elements approximately 9.0 Gyr ago. Therefore, the trajectory toward life not only required a large starting volume but also needed substantial development time.

While Earth’s ocean of 4 Gyr ago was large enough, its temperature [20] may not have allowed for a suitable memory chamber and the time duration to first life may not have been long enough. One alternative is a world similar to Europa that could have provided a more likely incubator for life and could have begun its prebiotic chemistry long before the solar system existed.

The paper is organized as follows: the calculation of the CHNOPS counts; the estimates of volumes needed to begin combinatoric chemistry; the regression of cell counts versus time; the extrapolation back in time to the basic chemical elements; discussion of the findings and examination of a theoretical world similar to Europa as an incubator of life long ago; and finally, a model of the pyramid of life that attempts to harmonize prebiotic chemistry and evolutionary processes.

## 2. Materials and Methods

### 2.1. Data

The analyses are based on the published report of the biomass distribution on Earth [19], which estimated 550 Gt of carbon distributed among all kingdoms of life. Approximately 450 Gt of carbon were assigned to plants. The remainder was distributed among other taxa: bacteria and archaea; protists and fungi; arthropods; other non-vertebrates (mollusks, nematodes, annelids); and vertebrates (birds, fish, wild mammals, livestock, humans). For this work, most plant carbon is considered non-living wood (90%) and vertebrates were divided into two groups: humans and all other non-human vertebrates.

To calculate cell numbers from the carbon content of various taxa, estimates of individual cell masses for the taxa were obtained. The broad range of cellular masses within taxa cannot be adequately addressed; therefore, basic orders-of-magnitude estimates were used and the smallest groups within each taxon were favored as likely early representatives during the course of evolution. Estimates for bacteria, archaea, and various eukaryotes were obtained from the supplemental tables of [21] and estimates of [22]. Living portions in trees, the dominant carbon repository, are primarily parenchymal cells. These are present in various shapes and sizes [23] but are significantly larger than vertebrate cells. The value of 3 × 10^−8^ g was chosen to approximate these cell types.

### 2.2. Analyses

Biomass estimates [19] were used to calculate the number of living cells within the various taxa. The assumptions for the calculations were that cells are 70% water and the dry mass of cells is 50% carbon. Therefore, the carbon in living cells is composed of 15% carbon. The amounts of non-living carbon in the various taxa were estimated as shown in footnote (b) of Table 1. Relative contributions of the CHNOPS elements were derived from Rossetto and Mansey [24] and were then used to calculate a mean molar mass of 12.8 g/mole for bacterial cells. This value was used to determine the mean number of CHNOPS elements needed to make 140 Gt of carbon-based biomass, yielding a value of 4.55 × 10^40^ units of mean elemental mass.

**Figure 1:**
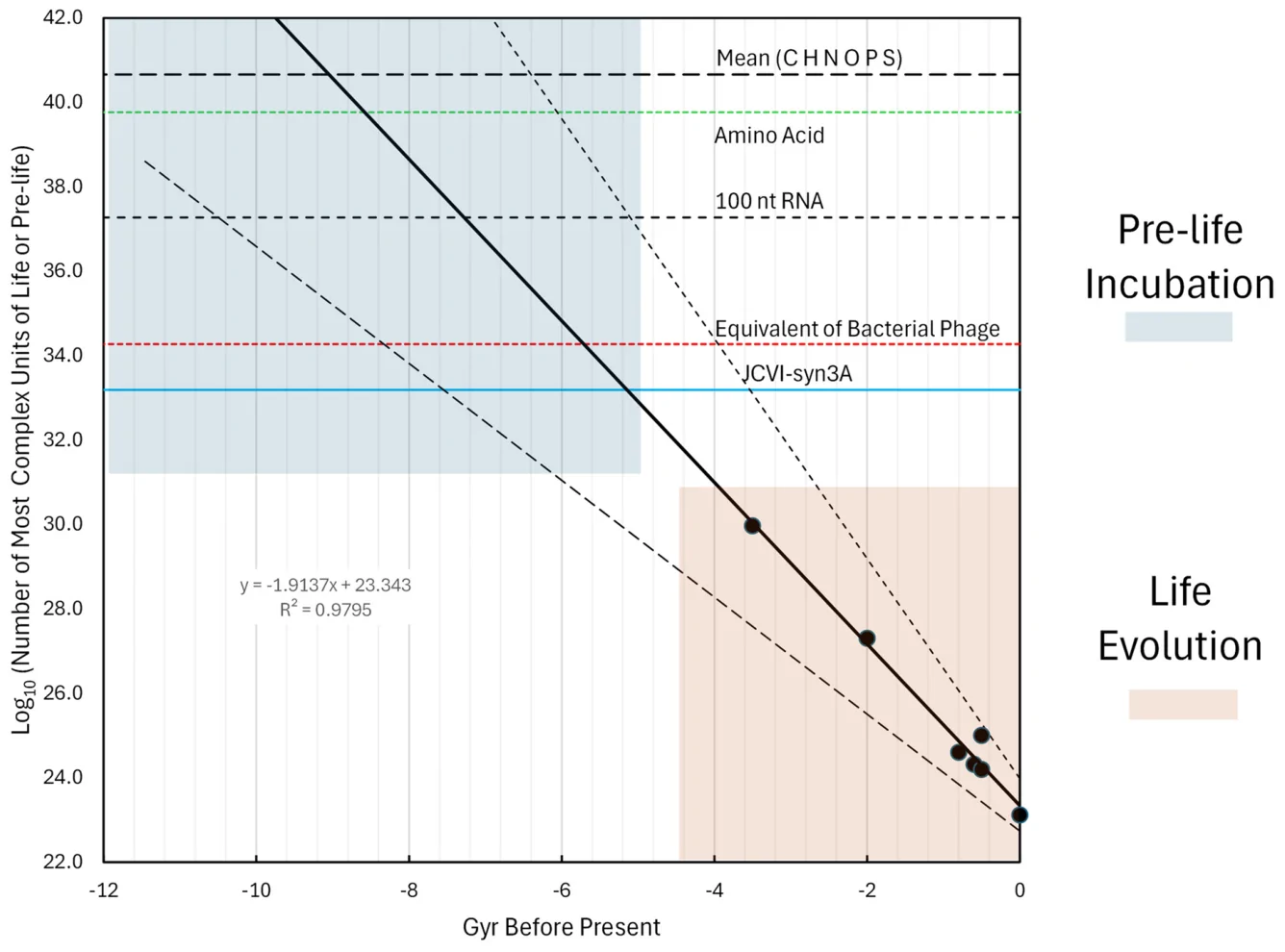
Log of cell numbers on Earth for most complex cell types for a given era, taken from Table 1. Regression (heavy black line) is extrapolated back in time until reaching the mean count of CHNOPS elements required to construct ~140 Gt of living cells. The regression line intersects other constructs that are on the development pathway to the origin of life. The confidence range, represented by the two dashed lines, is derived from a Monte Carlo simulation using simultaneous variation in both dimensions, as described in Appendix A. Orange block: time when Earth existed. Blue block: time preceding Earth and solar system.

Monte Carlo methods were used to determine the 95% confidence intervals around the regression in Figure 1. Sensitivity analyses for both the time and log cell count dimensions were performed to determine maximal variability in both dimensions, while maintaining values that are within known limits. The analyses are presented in Appendix A.

## 3. Results

### 3.1. Taxa Cell Masses and Cell Numbers

The distribution of carbon among taxa [19] is shown in the time = 0 column of Table 1. The total is equal to 140 Gt. The simplest assumption is that this represents the carrying capacity of Earth. The distribution of carbon over 3.5 Gyr from early bacteria and archaea to higher organisms was calculated with simple proportions so that the sum of each column matched the starting value of 140 Gt. The bold values in Table 1 represent the carbon associated with each taxon at its first appearance and first expansion to fill its niche. These values were used, together with cell sizes, to calculate the number of cells of these taxa when they first appeared. The log10 of those values are shown in the last column of Table 1.

The first goal of this paper is to determine the number of CHNOPS elements needed to build 140 Gt of carbon-based biomass. That value can be used to estimate a reaction and memory volume large enough to support the stochastic, combinatoric chemistry that could lead to life. This is presented in Section 4.2.

The second goal is to evaluate the continuous decrease in cell numbers as cells become more complex. This is shown with the log cell count versus time regression in Section 4.3. That regression was extrapolated to the log CHNOPS unit count, implicating a starting time for probiotic chemistry of 9 Gyr ago.

### 3.2. Estimates of Reaction Volume for CHNOPS

For combinatoric chemistry to begin from CHNOPS, there must be energy to drive the reactions and enough of each atomic element to create a suite of intermediates. Furthermore, there must be mechanisms to facilitate entropy imbalances, which will be determined by the geophysical environment [25,26,27]. Current life on Earth is limited by similar requirements, which can be referred to as Earth’s carrying capacity. For simplicity, it will be assumed that prebiotic chemistry had approximately equivalent energy and entropy constraints as current life on Earth, thus allowing for a focus on the availability of atomic elements as the main determinant of reaction and memory volume.

It is possible to calculate the absolute minimal volume using the mass of CHNOPS needed to construct life containing 140 Gt carbon. This minimum would be all the necessary elements mixed with water with the same ratio as a current living cell: i.e., 70% water and 30% dry weight of non-water molecules. This would be equivalent to one enormous cell. The count of mean CHNOPS units is 4.55 × 10^40^. Dividing this by Avogadro’s number, multiplying by the mean mass (12.8 g), and dividing by the fraction that is not water (0.3), and converting grams to liters, yields 3.2 × 10^15^ L. This is 2.4 × 10^−6^ of the total of Earth’s oceans, or 3235 cubic kilometers, which is about the size of Lake Huron in North America. This would be so packed with solutes and precipitates that prebiotic chemistry would be very difficult. While this provides a perspective on the minimum volume, it is desirable to calculate a more likely starting volume for prebiotic chemistry.

There are many approaches and much uncertainty when calculating a realistic reaction volume. In a soup of only atoms, the first question is whether there is one element that is in excess, thus leading to first-order kinetics. In this case, only a small fraction of that element would end up in the resulting molecules. First-order kinetics would be consistent with the log-linear relationship of Figure 1 for the biomass shares of life and the extrapolation to prebiotic chemistry.

Early Earth had abundant carbon and nitrogen after being reseeded from the outer solar system [28,29]. Nitrogen typically exists as N_2_ and has many characteristics of noble gases, as well as strong stability due to its internal triple bond. Carbon is much more reactive and rarely exists unbound to another element. Life, as we know it, is dominated by carbon; thus, it seems reasonable that carbon was in excess and other elements were rate-limiting.

The current concentration of carbon in the oceans is approximately 2 mM (ref [30], Table 1). On early Earth, it is estimated that there was 30–30,000-fold more carbon in the atmosphere and oceans [31]. For these calculations, a value of 100-fold was chosen as a low-end estimate. First-order kinetics are usually associated with a greater than 100-fold excess of one reactant; thus, a minimum of approximately 1% of the carbon present would end up in prebiotic molecules, driven by the rate-limiting concentrations of the other elements. The current carbon in Earth’s ocean associated with organic molecules is 2.6% of the total ocean carbon. Calculating the chamber size using this estimate of the fraction involved in prebiotic reactions yields a volume of approximately 0.024 of Earth’s oceans (Table 2), which would be about ten times the volume of the Mediterranean Sea. Using only carbon in living organisms, rather than all organic carbon, results in a much larger estimate of the chamber size.

Considering nitrogen, it may be less available. While it has about the same abundance in the solar system as carbon [32], it exists primarily as N_2_, which does not concentrate readily and is less reactive. Assuming that it existed in the same concentration during the prebiotic reactions of CHNOPS as it does now, and has a similar distribution in a rocky world, such as Earth, then approximately 2.4 × 10^19^ g [33] would be available in an ocean the size of Earth’s oceans. If all the nitrogen was available for reactions, then a chamber size of 0.36 of Earth’s oceans would be required (Table 2, footnote (b)). This volume would be proportionately higher if not all nitrogen was available for prebiotic chemistry.

These calculations are presented solely to show that reaction and memory chamber size can be very large, depending on assumptions. Additional work and discovery are required to narrow in on this important prebiotic chemistry constraint.

**Table 2:** Estimates of reaction + memory volumes for the origin of life.

Fraction of C or N Involved in Prebiotic Reactions	Based On	Chamber Size Liter H_2_O	Fraction of Earth’s Ocean
0.026 ^a^	Carbon	2.9 × 10^19^	0.022
0.00016 ^b^	Carbon	4.8 × 10^21^	3.7
1.0	Nitrogen ^c^	4.8 × 10^20^	0.36

^a^. Assuming that available carbon was 100-fold current ocean concentrations, yielding 0.2 M, and that the amount of carbon in prebiotic reactions was equal to the same fraction of organic carbon in current Earth’s oceans (1 × 10^18^ g [34]). ^b^. The same as (a), but using the fraction of carbon associated with life in current oceans (6 × 10^15^ g [35]). ^c^. Assuming that available nitrogen is equal to the current ocean concentration of 0.00065 M (calculated from the estimate of ocean N, dominated by N_2_ [33]).

### 3.3. Regression of Cell Numbers Versus Time

The log values from the last column of Table 1 were plotted versus the times of the first appearance of the taxa. This is shown in Figure 1, with the best-fitting linear regression line shown together with the 95% confidence interval calculated from the Monte Carlo simulation described in Materials and Methods and Appendix A. The regression line describes the steady progression to smaller counts for larger, more complex cells. Conversely, it describes the increase in cell counts for simpler cells in earlier eras. The regression can be extrapolated back in time to the horizontal line representing the mean number of starting CHNOPS elements required to construct ~140 Gt of living cells. The intersection with the CHNOPS line is at 9 Gyr before the present time, under the assumption of a stable slope. This is consistent with previous reports [2,3], and represents the second goal of this paper. Comments regarding the assumption of a stable slope are presented in Section 4.

A few of the constructs that would be on the path to cell formation are also shown in Figure 1. These horizontal lines are associated with the number of these molecules (or molecules of a similar size) that could be constructed from CHNOPS elements. They intersect the regression line close to the times reported for similar constructs in the complexity plots of [2]. A time for the appearance of a first, simple cell would be around 5 Gyr ago, slightly before our solar nebula formed. In this case, JCVI-syn3A [22,36,37,38] was chosen as a representative of a very early cell capable of survival and reproduction.

## 4. Elaboration and Discussion

### 4.1. Recap and Comments

The primary goal of this work is to estimate the starting volume for a combined reaction and memory chamber that would enable prebiotic chemistry to proceed toward the formation of life. Under the many assumptions of this paper, the volume was estimated to be very large. The results are based on the carbon biomass distribution [19] across various taxa of present-day Earth. The number of living cells was calculated and the total CHNOPS representing those cells was determined. Under the assumption that life began from a soup of CHNOPS elements in an undirected, combinatoric process, the volume needed to house the mean number of elements (~4.5 × 10^40^) could be a significant fraction of Earth’s total oceans. This represents the primary conclusion.

If cells are treated as basic units of life, then, by inference, the components of cells are a collection of biochemical units that enable life. These must lie along the general prebiotic trajectory between CHNOPS and life. To examine this, the logs of taxa cell numbers were plotted versus estimated times of first appearance to yield the trajectory of life on Earth. An apparent linear regression enabled extrapolation between life on Earth and CHNOPS unit counts as a first estimate for the timing of the origin of prebiotic chemistry. This yielded a value of approximately 9 Gyr before the present time. This is the secondary conclusion and is consistent with previous work [2,3].

#### 4.1.1. Analysis Assumptions and Variability

Figure 1 reveals a distinctive negative slope that arises from highlighting the progressive reduction in the numbers of the most complex cells with the time of first appearance. This is the opposite of the slope seen for complexity changes with time [2,3]. In both cases, the variability of data estimates must be examined. A study by Sharov and Gordon [3] yielded a regression on DNA length that was extrapolated to one base pair at 9.5 Gyr before the present time, using eight data points. They proposed panspermia as the logical conclusion. This was challenged by Marzban, Viswanathan, and Yurtsever [39] by using measurement error models. They concluded that a regression to a time within Earth’s history could not be ruled out. The analysis of much larger data sets over several complexity features, by Juckett [2], came to the same conclusion as that reached by Sharov and Gordon. Only minimal error analysis was performed on the regressions in that paper because of the strength of numbers for several complexity features and the consistent aggregate results of the regressions. Because of the small number of data points in Figure 1, a two-dimensional sensitivity analysis (Appendix A) was deemed necessary. It yielded the conclusion that assumptions regarding cell sizes, timing for the appearance of new lifeforms, and the implicit estimates for Earth’s current biomass distribution could affect the results but probably not the conclusions, i.e., that the 95% confidence intervals do not provide compelling evidence that prebiotic chemistry could have occurred on Earth. This is particularly true if that chemistry had been initiated by basic elements and proceeded randomly at the same rate that drives the random changes in the biochemistry underpinning evolution.

#### 4.1.2. Single-Slope Extrapolation Assumption

The conclusions of this paper and the previous one [2] depend on the validity of extrapolations past the data range. In general, extrapolations are only valid after being proven accurate for the system under study. They are often used in enzyme kinetics analyses that use empirical evidence for validity. In general, they are best used when they are predicted by a model and when extrapolating to either infinity or zero, where naturally occurring or experimental data are not available. Extrapolation is usually performed after a transformation (for example, the double reciprocals in Lineweaver–Burk plots [40] or the log–log plots of a power–law relationship). The log-linear plots used in this work and the previous work [2] are the linearization of an exponential process. Non-biological examples have demonstrated the utility of extrapolating beyond the data with a single slope [2]. Additional examples are shown in the Supplementary Materials related to US Library of Congress holdings extrapolated to the Gutenberg Bible, Figure S1, and the forward and reverse extrapolations of passenger vehicle velocities, Figure S2. These are not presented as evidence or proof that biology follows similar behavior, but that unexpected extrapolations exist and should at least be considered as the simplest explanation until proven otherwise.

An exponential decrease in reactant counts is predicted for first-order kinetics, as described in the Results; however, it also results from aggregation kinetics, as described by the rate equations of the Smoluchowski coagulation model [41,42]. These coagulation models apply for nebula consolidations into stellar systems, raindrops forming in atmospheres, and molecules aggregating within cells. Such kinetics would also be expected for the production of growing prebiotic constructs.

#### 4.1.3. Alternative to Single-Slope Extrapolation

If the regression of cell numbers and molecular units is a reasonable method with which to estimate both the progression of life and prebiotic chemistry, then any similar projection backward to CHNOPS would imply a much older Earth than current estimates. If the progression backward remains a linear extension of the regression of Figure 1, then Earth would need to be at least 9 Gyr old for life to form here. Since this is not possible, prebiotic chemistry and biotic chemistry must have occurred in different locations.

With a displacement in time and space, it is reasonable to assume that the regression slope could change due to the different environments of another world. However, it is anticipated that the number of CHNOPS would still need to be high to generate enough primordial cells to populate a stellar nebula for seeding worlds like Earth. Furthermore, the variation in element distribution in the galaxy and the variations in energy available on other worlds may not be large enough to dramatically change the prebiotic progression slope.

To avoid this displacement and enable life to form on Earth, prebiotic chemistry would need to proceed at a rate much faster than evolution. Some sigmoidal kinetics, similar to those describing polymerization [43], could provide a faster rate but it is incumbent on those who study prebiotic chemistry to demonstrate that this is possible.

#### 4.1.4. Carrying-Capacity Assumption

The Earth’s carrying capacity is assumed to be constant over the duration of life on Earth. This assumption is crude, but it has the benefit of enabling an estimate for the cell counts over time. The variables that could change this assumption include the variable that the Sun was 30% dimmer when Earth formed; however, there was probably more energy escaping from Earth’s interior to compensate for this. The availability of primary elements, particularly carbon, probably changed over time, with reseeding from the outer solar system. This is probably only important if all prebiotic chemistry took place on Earth. There is also an allometric relationship between organism size [44,45,46] and metabolism needs; thus, as organisms grow, they need proportionately less energy. However, using the slopes from [2], the allometric slope between the energy needed to build cells and cell mass is close to 1.0. The interplay of these variables is beyond the scope of this paper; therefore, a constant carrying capacity is favored for the first analysis.

Extending this carrying capacity to non-Earth prebiotic environments simply provides a “what if” examination of timing for the start of the chemical trajectory toward life as well as the volume constraints for this chemistry.

### 4.2. Europa-Style Moon as Possibility

A large volume, predicted by this analysis, could provide an adequate number of starting atoms; however, it does not guarantee an environment containing both reaction and memory components, nor enough time for reaction products to accumulate. It is anticipated that such an environment would need both hot reaction centers and cold memory spaces, combined with transport mechanisms and long development times for the accumulation of intermediates. It is not clear that early Earth could provide these conditions.

In addition, any prebiotic environment must provide protection from energy and molecular conditions that can destroy intermediates. This would include ionizing radiation, excessive heat, high or low pH values, excessive oxidant concentrations, or the presence of too many catalytic sites. Water environments, like those on the moons Europa, Ganymede, and Enceladus, could offer the needed protection and have already provided evidence for creating large organic molecules [47]. Similarly, planets with sufficient core heat or tidal heating, combined with an ice cap, could provide protection and stability for life to evolve. It does require that volatile elements (carbon and nitrogen) exist on such worlds by either being re-introduced after world formation, like Earth [48], or by being located beyond the evaporation zone. If our solar system is representative of other stars, then such moons and planets would have been prevalent throughout the universe’s history.

Early evidence regarding Europa makes it a suitable example of a world. It contains an ocean approximately twice the size of Earth’s, which is slightly above the freezing temperature of water at the depth it resides (see [49] and references therein). It has been estimated to slowly flow longitudinally. With heating from below, due to tidal stresses, it probably has meridional flows and upwelling gyres. Thus, it probably mixes in a manner that can bring water to both the underside of the ice cap as well as to the rocky sub-ocean surface, where heat escapes from the core, as magma flows, and through undersea vents. Such an environment can provide heat for reactions, minerals for catalysis, and cold water to provide a vast memory chamber for intermediates. With deep-sea venting, the water could be rich in dissolved CHNOPS elements. The 15–25 km thick ice cap would protect the water environment from the ionizing radiation of a planet similar to Jupiter, the local star, and cosmic rays. In such a world, prebiotic chemistry may be able to flourish, provided there is enough time.

A moon harboring an ocean on the order of Earth’s volume, following a path of development shown in Figure 1, would generate about 10^33^ cells, equivalent in size to JCVI-syn3A cells. If this moon’s contents were distributed into a nascent nebula by a cataclysm, then many of these cells would remain viable, depending on the nature of the disrupting event and whether they could be sequestered within large enough granules to be protected from cosmic rays. Our primordial solar nebula volume was estimated to be about one light year across, giving it a spherical radius of approximately 4.7 × 10^12^ km and a volume of ~4.4 × 10^38^ km^3^. This can be collapsed to a disk with an approximate radius of 50 astronomical units [50], which would be a sphere with a radius about 1000 times smaller, flattened into a disk with a thickness of perhaps 5–10% of the radius, yielding a crude volume on the order of 1 × 10^29^ km^3^. Therefore, every cubic kilometer could house 10^4^ cells, on average. If many stars developed from a larger nebula, then that would be a larger volume for one cell to occupy but is still very reasonable. As condensation of the nebula occurred, the cells would concentrate on accreting bodies and serve as seeds for planets and moons, when they were ready to nurture them [51].

### 4.3. Pyramid of Complexity

**Figure 2:**
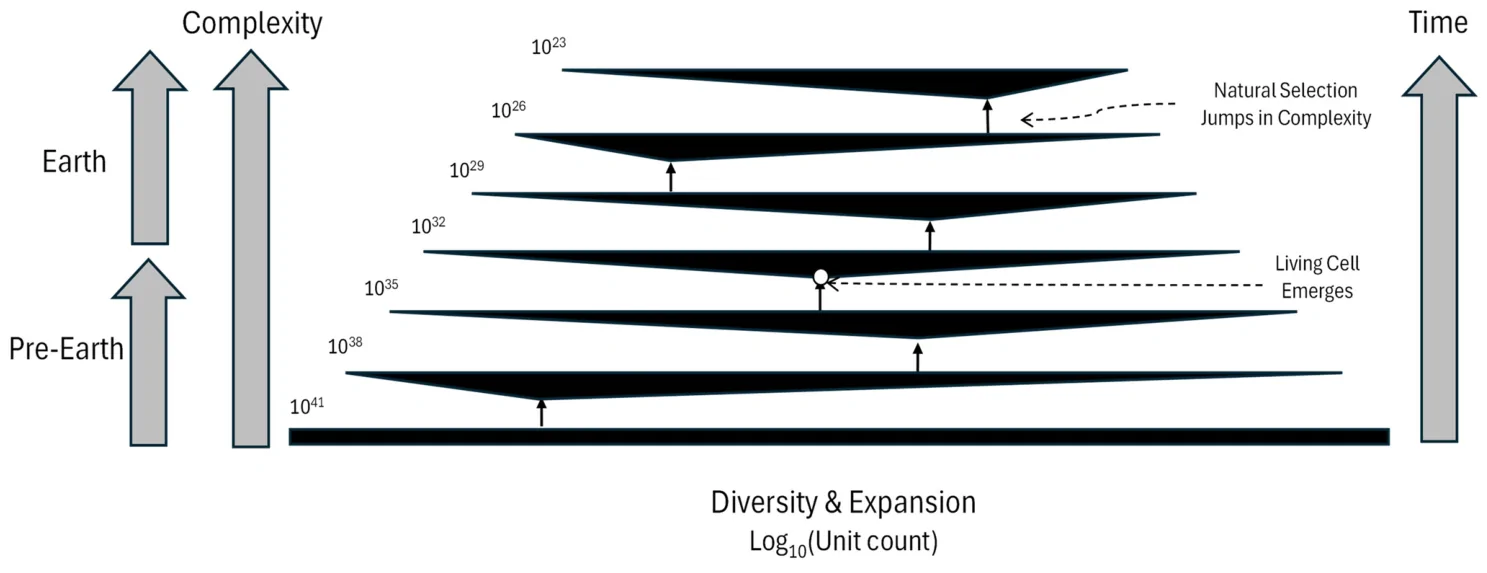
Pictorial representation of unit counts at growing levels of complexity from prebiotic chemistry to current life. With each jump, the number of more complex units expands until reaching the limit that the environment can support. That number decreases with each jump in complexity. Only a small set of jumps are shown in the figure for clarity. The values on the left of each bar represent the number of units decreasing in jumps of 1000-fold from the most numerous to the least numerous, taken from CHNOPS to humans in Figure 1. The lower regions represent prebiotic chemistry that may occur on a pre-Earth location before the Earth formed. At some time and level of complexity, an encapsulated living unit (cell) emerges. From that point upward, the units are cells. The time span is anticipated to be 8–10 Gyr.

The cartoon graphic of Figure 2 is an attempt to combine complexity growth plus the results of this analysis with the evolutionary concepts of natural selection, population expansion, and diversity. The local universe is not large enough to contain all the possible combinatoric outcomes of chemistry and biochemistry, particularly when counting all the unique sequences that can be constructed from long polymers like polynucleotides and amino acid chains. Therefore, it is proposed that complexity grows in jumps, with each level defining a new set of combinatoric possibilities.

The small vertical arrows of Figure 2 denote the sudden mutations (or new chemical entities) that lead to increases in complexity, hallmarked by lower entropy, higher efficiency, dynamic stability, fecundity, etc. For prebiotic chemistry, the complexity jumps may be indicative of larger molecular constructs, greater molecular stability, the appearance of enzymatic pockets, non-covalent binding, the beginning of networks, etc. Each of these jumps is a random event that probably is described by extreme value theory [52] and thus is tied to fluctuations around equilibria. Once a jump occurs, the new entity types can expand in number and diversity within a given complexity level. The expansion is limited by the amount of available energy and the capacity to absorb the entropy imbalances, i.e., the greater the complexity, the smaller the population with that complexity. Ultimately, complexity jumps enable new constructs and diversity. Subsequently, diversity and expansion lock in complexity by providing a buffer against all the forces that can degrade the new chemical and biochemical constructs.

### 4.4. Final Comments and Conclusion

The purpose of Figure 2 is to suggest that prebiotic chemistry and the biochemistry of life follow the same rules of nature and, thus, are a natural consequence of a developing universe. Both chemistry and biochemistry can randomly yield novel constructs in a stepwise fashion, provided there is enough working material, space, and time. Most importantly, there must be a mass balance from bottom to top to maximize the likelihood of the progression from atoms to life. Without this mass balance, there would need to be a very large ensemble of worlds, with each harboring only enough reactive chemical mass to enable narrow prebiotic pathways that mostly lead to dead ends. Is Earth a world with a lucky combination of trajectories that led to life or is it one of a constellation of worlds that simply nurtured early life into advanced life?

Steven Gould [53] considered that a Left Wall of Minimal Complexity explained the random walk of evolution from bacteria to more complex cells. It might be argued that each transition in complexity shown in Figure 2 generates a new local minimal complexity that is the basis for new random walks of evolution. Thinking in these terms, the origin of life has the characteristics of a ratchet that locks advancement in place by expansion and diversity within each new complexity level. This makes it resistant to cataclysmic extinction events, as long as there are a few representatives remaining within a complexity level.

Prebiotic chemistry is not just an Earth story; it is a universe story. As suggested by Figure 1, the universe has had enough time to enable prebiotic chemistry to evolve through combinatoric processes, leading to life that can seed nearby nebula for further development. Prebiotic chemistry in some early worlds may not lead to life, while life may form in others. Some of that life could easily be totally different from what we know, but it may have about the same level of complexity as what we know [3,54] because complexity takes time to build. The challenge for the field of prebiotic chemistry is to determine how life can form, in whatever shape it takes.

In conclusion, for life to have originated on Earth, a series of highly improbable events needed to have occurred to reach the complexity of a living organism. If life began through stochastic processes, it needed to have occurred within a volume that included reaction centers and memory storage compartments. The purpose of this paper is to raise this issue and stimulate discussion on how to address it.

(*Note added in support of findings*). The slope of Figure 1 was not addressed in this paper, but it may have an origin in thermodynamic properties. In a previous paper [2], the various complexity slopes spanned both energy-related and entropy-related features of a living cell. In particular, the total Gibbs free energy [22] to build the components of a cell was one of the complexity features examined. This represents the simplest form of structure, i.e., a bag of parts. The prebiotic trajectories for the origin of life have assembled these parts into interacting networks and function-determining structures, together with memory molecules to record these assemblies. This added complexity demands a further lowering of entropy than that needed to construct the random bag of parts. Furthermore, with each jump in complexity, entropy must decrease more. The complexity features of DNA length, gene number, and protein–protein binding counts represent such entropy-related structures; when their slopes are combined with the slopes from the energy to build cell parts, the negative of the slope of Figure 1 can be approximated. From this, it may be inferred that the reduced number of more complex cells is a direct result of increasing energy and entropy requirements, which may be empirically examined. This will be the subject of a future report, which will address some of the thermodynamic constraints that span the origin and maintenance of life.

## Figures and Tables

**Table 1:** Compilation of cellular carbon and cell number distribution over time.

	Gyr Before Present Time								
	−3.5	−2	−0.8	−0.6	−0.5	−0.5	0 ^a^	Values for Most Complex Taxa in a Given Era	
Taxa	Distribution of Cellular Carbon (Gt) ^b^							Cell Mass (g) ^c^	Log_10_(Cell #) ^d^
Bacteria	**140.00**	115.91	114.91	114.40	77.42	77.03	77.00	1.0 × 10^−12^	30.0
Fungi		**24.09**	23.88	23.77	16.09	16.01	16.00	8.0 × 10^−11^	27.3
Arthropods			**1.21**	1.20	0.81	0.81	0.81	2.0 × 10^−9^	24.6
Other invertebrates				**0.63**	0.42	0.42	0.42	2.0 × 10^−9^	24.3
Plants					**45.25**	45.02	45.00	3.0 × 10^−8^	25.0
Vertebrates						**0.71**	0.71	3.0 × 10^−9^	24.4
Humans							**0.06**	3.0 × 10^−9^	23.3

^a^. Present day. ^b^. Adjusted to remove non-cellular carbon content. Bacteria and fungi are assumed to be 100% cellular; arthropods and other invertebrates are assumed to be 80% cellular; plants are assumed to be 10% cellular; and vertebrates are assumed to be 85% cellular. These are derived from estimates of animal shells, wood, and bones in the respective taxa. ^c^. Approximate cell masses for the various taxa were obtained from sources described in Materials and Methods. ^d^. The logarithm of cellular carbon is in grams (using bolded values) divided by cell mass, assuming that carbon is 15% of the cell mass.

**Figure A1:**
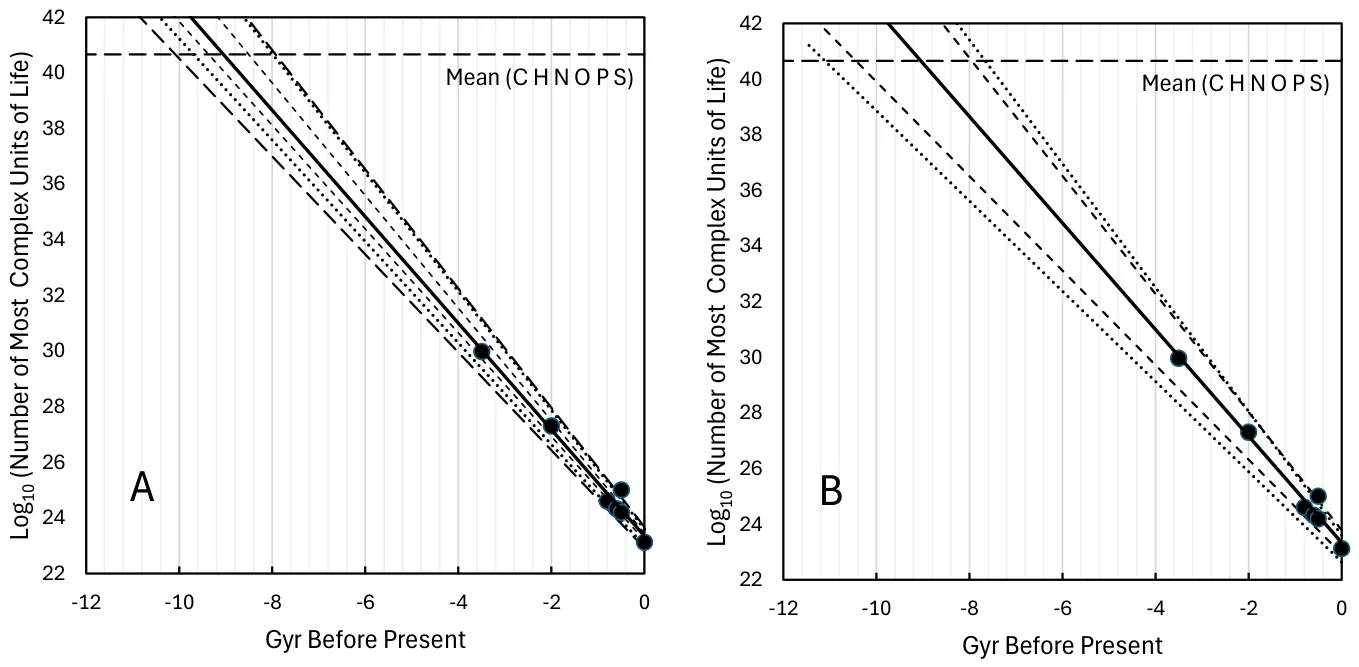
The 95% confidence regression ranges for variations of variable ***B*** (panel (**A**)); and variable ***M*** (panel (**B**)). The magnitude of the random variations and the line types are given in Table A1. Data points from Figure 1 of the main text are shown as solid dots, with the regression shown as a solid line.

**Figure A2:**
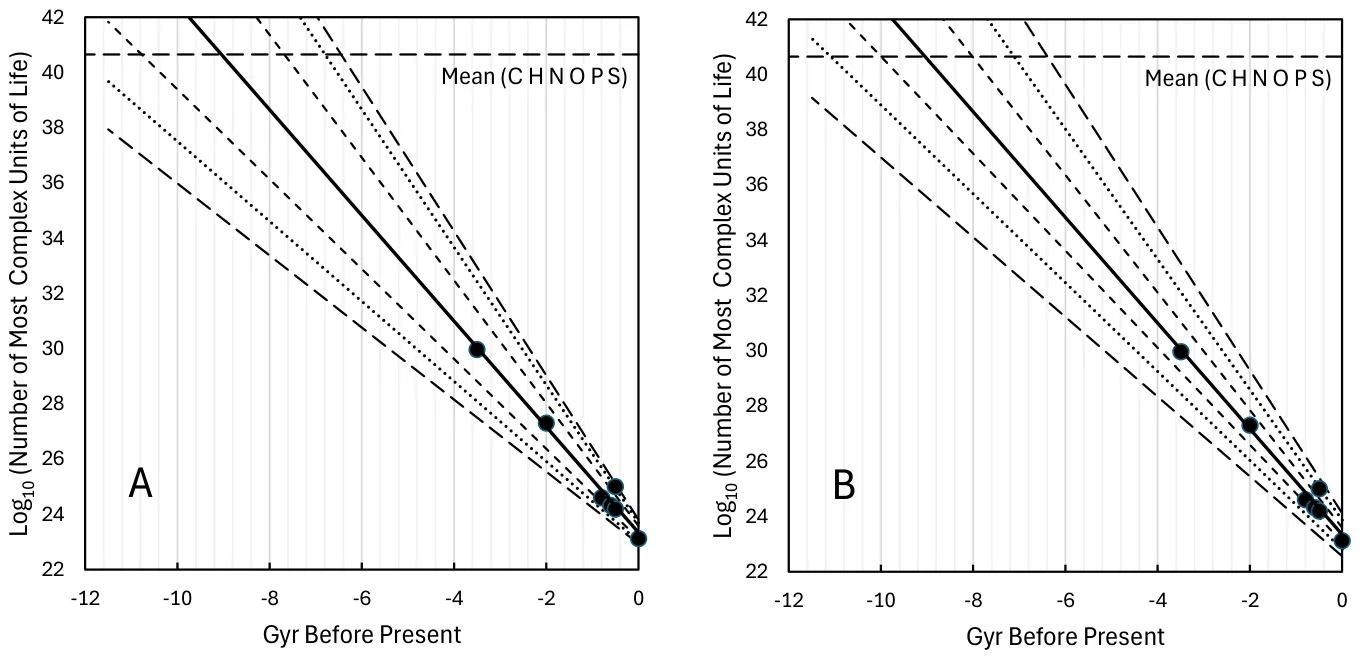
The 95% confidence regression ranges for variations of variable *t* (panel (**A**)); and log[***C***] (panel (**B**)). The magnitude of the random variations and the line types are given in Table A2. Data points from Figure 1 of the main text are shown as solid dots, with the regression shown as a solid line.

## Data Availability

All source data used in this paper were previously published by other authors, as detailed in the text.

## Supplementary Materials

The following supporting information can be downloaded at: https://www.mdpi.com/article/10.3390/life16101708/s1, Figure S1: Extrapolation of US Library of Congress holdings to Gutenberg Bible, and Figure S2: Extrapolation of passenger vehicle velocities from bicycles to spacecraft. References [2,55].

## Abbreviations

The following abbreviations are used in this manuscript:

Gyr	Giga years
CHNOPS	Carbon Hydrogen Nitrogen Oxygen Phosphorus Sulfur
JCVI-syn3A	Human constructed minimal cell [37]

## Appendix A

The 95% confidence interval around the regression slope in Figure 1 is the result of a Monte Carlo analysis using normally distributed random variates to represent time and log[cell number] estimates for each of the taxa. In this appendix, a sensitivity analysis is described that isolates the variability caused by each component and provides the rationale for the choice depicted in Figure 1.

A regression of log cell number versus time-of-first-appearance can be generically defined by a function:

**Regression** [*x*, *y*], where *x* and *y* are one-dimensional arrays of values.

This can return values for slope (*a*), intercept (*b*), and indicators of goodness of fit such as R^2^. The values in *y* are the log[cell counts], where cell counts (***C***) are derived from estimates of carbon biomasses, ***B***, (in Gt) and cellular masses, ***M***, (in g) for various taxa, *τ*, corrected by the 15% carbon content in living organisms, and units corrected with the 10^15^ conversion of Gt to gram. This yields:

***C***τ = ***B****τ* 10^15^/***M****τ*/0.15,

and

*yτ* = log[***C****τ*].

The values in *x* are given by estimates of time, ***t****τ*, (in Gyr) for the approximate first appearance of each taxa group selected.

### Appendix A.1. Local Sensitivity Analysis

Each of the three components, ***B****τ*, ***M****τ*, and ***t****τ*, can be varied independently, while holding the others constant, to determine their separate effects on the confidence interval shown in Figure 1. The components ***B*** and ***M*** appear within the logarithm and require large percentage variations to affect the log values. Such variations can generate significant numbers of samples with negative values that contain no meaning. This is shown in Table A1, which demonstrates the number of valid cases, out of 10,000, for a few choices of random variation. The variations with valid results yield only narrow 95% confidence ranges around the regression line, as shown in Figure A1.

**Table A1:** Single-variable Monte Carlo simulations of variables ***B*** and ***M***.

Variable	Coef Var (%) for Random Variate †	Valid Cases	Figure A1 Line Type
Biomass, ***B***	25	9997	- - - - -
	50	8526	• • • • •
	75	5042	− − − −
Cell masses, ***M***	100	3003	- - - - -
	250	525	• • • • •

† Random variates were chosen from a normal distribution with mean equal to the cell count for a given taxon and standard deviation given by the coefficient of variation listed.

Therefore, variations in the log scale were chosen to address the problem of the low valid case count in the Monte Carlo analysis. This also allowed for realistic variations in cell masses, ***M***, which are only order-of-magnitude estimates. Each of the 10,000 cases allowed for random variation independently on ***t*** and the log cell number for each taxon. Examples of results for this approach are shown in Table A2 and Figure A2. Although not shown, the lowest valid case count among these examples was 9962. For the random variations shown in Table A2, the effects on prokaryote time of appearance and cell numbers are shown as ranges [−2sd, +2sd], where sd is standard deviation. For the time dimension, a coefficient of variation of 15% for the random number distribution was deemed appropriate because it generated a range that spanned 2.45 to 4.45 Gyr. Any larger coefficient of variation and the range would include times before Earth existed. For the log cell number dimension, the coefficient of variation of 2% was deemed appropriate because it generated a range of 2.4 orders of magnitude for prokaryote mass and somewhat smaller values for other taxa. Most of this range would result from the uncertainties in the estimates of ***M***, since ***B*** had an error range no larger than a factor of 2 [19]. It is assumed that estimates of ***M*** would not be off by more than ± 1.2 orders of magnitude. Therefore, these two choices were used simultaneously to generate the 95% confidence intervals of Figure 1.

**Table A2:** Single-variable Monte Carlo simulation results for *t* and log[***C***].

Variable	Coef Var (%) for Random Variate †	Variation Around 3.5 Gyr		Figure A2 Line Type
		−2 sd	+2 sd	
Time of taxa appearance, *t*	10	2.80	4.20	- - - - -
	15	2.45	4.55	• • • • •
	20	2.10	4.90	− − − −
		**Order of Magnitude Range** ‡ **from** **−2 sd to +2 sd**		
Log[*C*]	1	1.2		- - - - -
	2	2.4		• • • • •
	3	3.6		− − − −

† Random variates were chosen from a normal distribution with mean equal to the time of appearance for each taxon, as shown in Table 1 of the main text, and standard deviation given by the coefficient of variation listed. ‡ This is the range in the *y* dimension for the data point representing prokaryotic organisms.

### Appendix A.2. Software

Analyses were performed in Excel^®^, running 10,000 replicates for each case using the **What-IF Analysis** capability combined with the **Data Table** function. The SLOPE, INTERCEPT, and RSQ functions were used to yield parameter values of the linear regression for each of the 10,000 replicates and each of the targets of variation. The distributions of the slopes and intercepts exhibited robust lognormal shape. These were best-fit to the lognormal function using the SOLVER add-in within Excel^®^. The resulting means and non-symmetrical standard deviations derived from the lognormal fits were used to examine the 95% confidence intervals around the linear regressions.

(Note: Excel^®^ was chosen for these analyses because of its relatively large availability and its robust capabilities for these calculations. It gives others the greatest chance for reproducing the results. Such calculations can be performed in various platforms, such as Matlab^®^, NumPy in Python, R, etc., but I did not choose to write these programs).

## References

[1] Cleaves H.J. (2013). Prebiotic Chemistry: Geochemical Context and Reaction Screening. Life.

[2] Juckett D.A. (2026). Can the Timing of the Origin of Life Be Inferred from Trends in the Growth of Organismal Complexity?. Life.

[3] Sharov A.A., Gordon R. (2013). Life Before Earth. arXiv.

[4] Martin W., Baross J., Kelley D., Russell M.J. (2008). Hydrothermal Vents and the Origin of Life. Nat. Rev. Microbiol..

[5] Baross J.A., Hoffman S.E. (1985). Submarine Hydrothermal Vents and Associated Gradient Environments as Sites for the Origin and Evolution of Life.

[6] Lyons T.W., Krishnamurthy R., Rogers K.L., Williams L.D. (2026). Introduction to the Special Collection: Early Earth Environments and the Origins of Life. Astrobiology.

[7] Dodd M.S., Papineau D., Grenne T., Slack J.F., Rittner M., Pirajno F., O’Neil J., Little C.T.S. (2017). Evidence for Early Life in Earth’s Oldest Hydrothermal Vent Precipitates. Nature.

[8] Kauffman S.A. (2011). Approaches to the Origin of Life on Earth. Life.

[9] Cinquemani S.M., Lutz R.A. (2026). Deep-Sea Hydrothermal Vent and Impact-Generated Hydrothermal Vent Systems: Insights into the Origin of Life. J. Mar. Sci. Eng..

[10] Szostak J.W. (2016). On the origin of life. Medicina.

[11] Schrum J.P., Zhu T.F., Szostak J.W. (2010). The Origins of Cellular Life. Cold Spring Harb. Perspect. Biol..

[12] Andrulis E.D. (2011). Theory of the Origin, Evolution, and Nature of Life. Life.

[13] Damer B., Deamer D. (2020). The Hot Spring Hypothesis for an Origin of Life. Astrobiology.

[14] Camprubí E., de Leeuw J.W., House C.H., Raulin F., Russell M.J., Spang A., Tirumalai M.R., Westall F. (2019). The Emergence of Life. Space Sci. Rev..

[15] Vázquez-Salazar A. (2026). Complex and Messy Prebiotic Chemistry: Obstacles and Opportunities for an RNA World. Life.

[16] Domagal-Goldman S.D., Wright K.E., Adamala K., De La Rubia L.A., Bond J., Dartnell L.R., Goldman A.D., Lynch K., Naud M.E., Paulino-Lima I.G. (2016). The Astrobiology Primer v2.0. Astrobiology.

[17] Stüeken E.E., Anderson R.E., Bowman J.S., Brazelton W.J., Colangelo-Lillis J., Goldman A.D., Som S.M., Baross J.A. (2013). Did Life Originate from a Global Chemical Reactor?. Geobiology.

[18] Bowman J.C., Goldenfeld N., Rogers K.L., Petrov A.S., Williams L.D. (2026). Rethinking the Last Universal Common Ancestor of Life: Network Convergence and the Root of the Tree. Astrobiology.

[19] Bar-On Y.M., Phillips R., Milo R. (2018). The Biomass Distribution on Earth. Proc. Natl. Acad. Sci. USA.

[20] Ueda H., Shibuya T. (2021). Composition of the Primordial Ocean Just after Its Formation: Constraints from the Reactions between the Primitive Crust and a Strongly Acidic, Co2-Rich Fluid at Elevated Temperatures and Pressures. Minerals.

[21] Gonzalez-de-Salceda L., Garcia-Pichel F. (2021). The Allometry of Cellular DNA and Ribosomal Gene Content among Microbes and Its Use for the Assessment of Microbiome Community Structure. Microbiome.

[22] Ortega-Arzola E., Higgins P.M., Cockell C.S. (2024). The Minimum Energy Required to Build a Cell. Sci. Rep..

[23] Crang R., Lyons-Sobaski S., Wise R. (2019). Plant Anatomy A Concept-Based Approach to the Structure of Seed Plants.

[24] Rossetto D., Mansy S.S. (2022). Metals Are Integral to Life as We Know It. Front. Cell Dev. Biol..

[25] Michaelian K. (2011). Thermodynamic Dissipation Theory for the Origin of Life. Earth Syst. Dyn..

[26] Vanchurin V., Wolf Y.I., Koonin E.V., Katsnelson M.I. (2022). Thermodynamics of Evolution and the Origin of Life. Proc. Natl. Acad. Sci. USA.

[27] Tuck A.F. (2023). Natural Selection and Scale Invariance. Life.

[28] Gail H.P., Trieloff M. (2017). Spatial Distribution of Carbon Dust in the Early Solar Nebula and the Carbon Content of Planetesimals. Astron. Astrophys..

[29] Marty B., Alexander C.M.O.D., Raymond S.N. (2013). Primordial Origins of Earth’s Carbon. Rev. Mineral. Geochem..

[30] Devries T. (2026). The Ocean Carbon Cycle. Annu. Rev. Environ. Resour..

[31] Dismukes G.C., Klimov V.V., Baranov S.V., Kozlov Y.N., Dasgupta J., Tyryshkin A. (2001). The Origin of Atmospheric Oxygen on Earth: The Innovation of Oxygenic Photosynthesis. Proc. Natl. Acad. Sci. USA.

[32] Palme H. (2003). Solar System Abundances of the Elements. Treatise on Geochemistry.

[33] Li Y. (2024). The Origin and Evolution of Earth’s Nitrogen. National Science Review.

[34] Falkowski P., Scholes R.J., Boyle E., Canadell J., Canfield D., Elser J., Gruber N., Hibbard K., Högberg P., Linder S. (2000). The Global Carbon Cycle: A Test of Our Knowledge of Earth as a System. Science.

[35] Bar-On Y.M., Milo R. (2019). The Biomass Composition of the Oceans: A Blueprint of Our Blue Planet. Cell.

[36] Hutchison C.A., Chuang R.Y., Noskov V.N., Assad-Garcia N., Deerinck T.J., Ellisman M.H., Gill J., Kannan K., Karas B.J., Ma L. (2016). Design and Synthesis of a Minimal Bacterial Genome. Science.

[37] Breuer M., Earnest T.M., Merryman C., Wise K.S., Sun L., Lynott M.R., Hutchison C.A., Smith H.O., Lapek J.D., Gonzalez D.J. (2019). Essential Metabolism for a Minimal Cell. eLife.

[38] Moger-Reischer R.Z., Glass J.I., Wise K.S., Sun L., Bittencourt D.M.C., Lehmkuhl B.K., Schoolmaster D.R., Lynch M., Lennon J.T. (2023). Evolution of a Minimal Cell. Nature.

[39] Marzban C., Viswanathan R., Yurtsever U. (2014). Earth before Life. Biol. Direct.

[40] Segel I.H. (1975). Enzyme Kinetics.

[41] Wattis J.A.D. (2006). An Introduction to Mathematical Models of Coagulation-Fragmentation Processes: A Discrete Deterministic Mean-Field Approach. Phys. D Nonlinear Phenom..

[42] Szała-Mendyk B., Drajkowska A., Molski A. (2023). Modified Smoluchowski Rate Equations for Aggregation and Fragmentation in Finite Systems. J. Phys. Chem. B.

[43] Achilias D.S. (2007). A Review of Modeling of Diffusion Controlled Polymerization Reactions. Macromol. Theory Simul..

[44] West G.B., Woodruff W.H., Brown J.H. (2002). Allometric Scaling of Metabolic Rate from molecules and Mitochondria to Cells and Mammals. Proc. Natl. Acad. Sci. USA.

[45] DeLong J.P., Okie J.G., Moses M.E., Sibly R.M., Brown J.H. (2010). Shifts in Metabolic Scaling, Production, and Efficiency across Major Evolutionary Transitions of Life. Proc. Natl. Acad. Sci. USA.

[46] White C.R., Alton L.A., Bywater C.L., Lombardi E.J., Marshall D.J. (2022). Metabolic Scaling Is the Product of Life-History Optimization. Science.

[47] Postberg F., Khawaja N., Abel B., Choblet G., Glein C.R., Gudipati M.S., Henderson B.L., Hsu H.W., Kempf S., Klenner F. (2018). Macromolecular Organic Compounds from the Depths of Enceladus. Nature.

[48] Pathak D., Dasgupta R. (2025). The Existing Frameworks of Delivery of Major Volatiles and the Feasibility of Mars-Mass Planetary Embryos as the Major Volatile Contributors to Bulk Silicate Earth. Astrobiology.

[49] Ashkenazy Y., Tziperman E. (2021). Dynamic Europa Ocean Shows Transient Taylor Columns and Convection Driven by Ice Melting and Salinity. Nat. Commun..

[50] Kretke K.A., Levison H.F., Buie M.W., Morbidelli A. (2012). A Method to Constrain the Size of the Protosolar Nebula. Astron. J..

[51] Wallis M.K., Wickramasinghe N.C. (2004). Interstellar Transfer of Planetary Microbiota. Mon. Not. R. Astron. Soc..

[52] Castillo E. (1988). Extreme Value Theory in Engineering.

[53] Gould S.J. (1994). The Evolution of Life on Earth. Sci. Am..

[54] Petri V. (2025). Robust Exponential Complexity Growth in the Universe. J. Big Hist..

[55] Bejan A., Charles J.D., Lorente S. (2014). The Evolution of Airplanes. J. Appl. Phys..

