# Supplementary material for: Estimates for the Prebiotic Reaction Volume Necessary for the Origin of Life
Source: Life (Basel). 2026 Oct 12;16(10):1708. doi: 10.3390/life16101708 (PMC13706395; doi:10.3390/life16101708)
Supplement: Supplementary file 1 [file life-16-01708-s001.zip › life-4550636-supplementary.pdf]

life

MDPI

Supplement to:

Estimates for the Prebiotic Reaction Volume Necessary for the Origin of Life

David A. Juckett

The complexity extrapolations of the earlier paper (Juckett, 2026) were all to subunits. This was also seen in Moore's law plot of microprocessors and computer memory, both extrapolating down to a single transistor. Two additional modern analogies are shown in this Supplement. One is the extrapolation of the US Library of Congress holdings backward in time to a single printed page (subunit) of the Gutenberg Bible and the other contains extrapolations both backward and forward in time for velocities of passenger carrying craft, from tandem bicycles to rockets. Examples from modern case studies are not proof of principle, they are only a collection of cases for which extrapolation beyond the data is a valid exercise. In particular, these examples show extrapolations to subunits. The accuracy of these extrapolations give reason to at least consider that exponential processes can extend back into the times of the early universe for processes that take a long time to develop, such as the origin of life.

Gutenberg Extrapolation

The US Library of Congress holdings were given in the Supplement file of the previous paper [2]. In the figure below, the log10 of these entries is plotted versus year and the regression was extrapolated back to the year 1400 CE. The Gutenberg bible was printed in 1455 and contained 1288 pages. (https://en.wikipedia.org/wiki/Gutenberg_Bible, accessed 8-10-2026). The date of printing and the fraction of the book represented by a single page are shown as vertical and horizontal lines in the figure, respectively. The regression lines, with and without the intersection of these lines reveal almost identical slopes, intercepts, and regression coefficients. A single page represents a basic subunit of the holdings since the library contains about 39M books, 70M documents, 25M music scores, plus approximately 40M other holdings. What they all have in common is a single page as a fundamental subunit. It is likely that if each of the holdings were broken down into individual pages, or the equivalent, then the points on the plot would shift up by a constant fraction in the log space thus changing the slope but not the intercept.

 https://doi.org/10.3390/life16101708

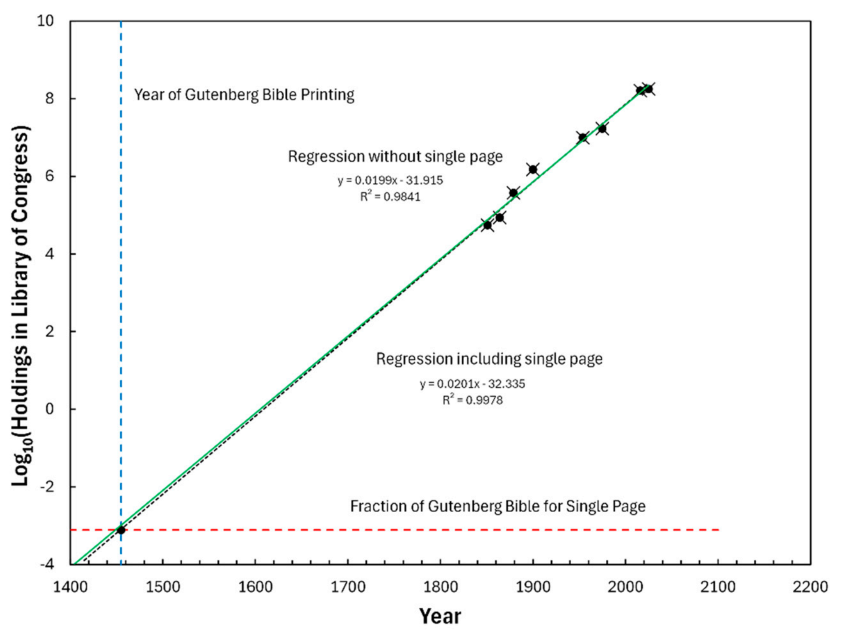

Figure S1. Plot of logarithm of holding of the US Library of Congress versus calendar year. Data was described in text. Two regressions were performed; on included the data point for the Gutenberg Bible page and the other was without this data point.

Commercial Passenger Craft Velocities

Data on various aspects of commercial airlines were provided in the supplemental files of Bejan, Charles, and Lorente, 2014. They showed an allometric scaling relationship between log[velocity of airplanes] and log[mass]. This also included flying, running, and swimming animals and insects. Their data for commercial airplanes is plotted below as black dots and green x's using log Mach number versus Year CE. This yields an exponential increase in velocity over time. The regression line is only for the black dots since airplanes built after approximately 1960 CE were required to fly below the speed of sound except the Concorde. The regression line was extended backward and forward in time to examine if other data fell on the line. All data is restricted to vehicles that can carry a passenger with the only exception of the 1903 first Wright flyer.

Passengers traveling to low Earth orbit has only become common since about 2020 CE. The blue circles are examples for space tourism commercial flights to low Earth orbit that was obtained from: https://orbitalradar.com/missions/space-tourism (accessed 8-10-2026). To fly to the moon requires reaching escape velocity. This plot predicts that someone will offer a flyby as early as 2029 for paying customers. Similarly, a flyby of Mars could be in the offing as soon as 2045 if the regression line is predictive. The extrapolation into the past intersects with the patenting of the tandem bicycle, which can be considered one of the subunits of all modern passenger ground vehicles.

https://doi.org/10.3390/life16101708

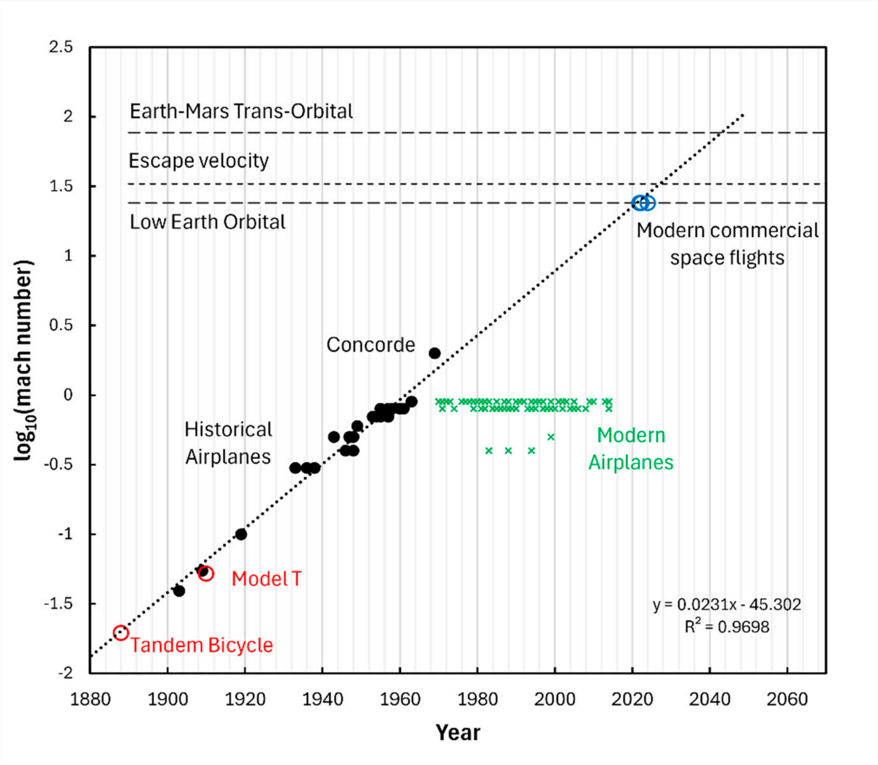

Figure S2. Plot of the logarithm of passenger vehicle speed (as mach number) versus calendar year. Regression included all black data points and the two red circles. Blue circles are dates and mach numbers for pay-to-ride astronauts to low Earth orbit. Selected space travel velocities are shown as horizontal dashed lines.

REFERENCES

Juckett, D. Can the Timing of the Origin of Life Be Inferred from Trends in the Growth of Organismal Complexity? *Life*, **2026**, *153* /doi.org/10.3390/life16010153

Bejan, A.; Charles, J.D.; Lorente, S. The Evolution of Airplanes. *J. Appl. Phys.* **2014**, *116*; doi: 10.1063/1.4886855.

Disclaimer/Publisher's Note: The statements, opinions and data contained in all publications are solely those of the individual author(s) and contributor(s) and not of MDPI and/or the editor(s). MDPI and/or the editor(s) disclaim responsibility for any injury to people or property resulting from any ideas, methods, instructions or products referred to in the content.
